# Artificial Intelligence-Enhanced Cardiac Magnetic Resonance in Hypertrophic Cardiomyopathy: Diagnosis, Phenotypic Characterization, and Risk Prediction

**DOI:** 10.3390/diagnostics16203328

**Published:** 2026-10-13

**Authors:** Yu-Jie Xiang, Yu-Cong Zheng, Ju Han, Qian-Mo Wei, Ting Yang, Rui Li

**Affiliations:** 1 Department of Radiology, Affiliated Hospital of North Sichuan Medical College, Nanchong 637000, China; jadex2002@163.com (Y.-J.X.); hanju0417@163.com (J.H.); for4shonee@163.com (Q.-M.W.); yangting023@163.com (T.Y.); 2 Department of Radiology, Tsinghua University Hospital, Tsinghua University, Beijing 100084, China; zhengcongcong1211@gmail.com; 3 Sichuan Key Laboratory of Medical Imaging, Nanchong 637000, China

**Keywords:** hypertrophic cardiomyopathy, cardiac magnetic resonance, artificial intelligence, radiomics, machine learning, deep learning, late gadolinium enhancement, risk stratification

## Abstract

Hypertrophic cardiomyopathy (HCM) is a genetically and phenotypically heterogeneous myocardial disease associated with a broad spectrum of clinical outcomes, including heart failure, atrial fibrillation, ventricular arrhythmias, and sudden cardiac death. Cardiac magnetic resonance (CMR) plays a central role in the comprehensive assessment of HCM by enabling integrated evaluation of myocardial morphology, ventricular and atrial function, tissue characterization, myocardial deformation, and longitudinal disease progression within a single comprehensive imaging modality. However, conventional CMR interpretation remains limited by manual post-processing, interobserver variability, sequence-dependent measurements, and reliance on a relatively restricted set of predefined imaging parameters. These limitations may hinder the full exploitation of the rich multidimensional information contained in CMR datasets. Artificial intelligence (AI) approaches, particularly machine learning and deep learning, together with radiomics-based quantitative image analysis, provide new opportunities to extend the diagnostic and prognostic value of CMR to extract high-dimensional and quantitative information from cine imaging, late gadolinium enhancement, parametric mapping, and feature-tracking-based deformation analysis. Emerging evidence suggests that AI-enhanced CMR may facilitate automated image analysis, improve diagnostic classification, support differentiation of HCM from phenocopies, enable tissue- and deformation-based phenotypic characterization, and enhance individualized risk prediction. Although several studies have included independent or external testing, many remain retrospective, rely on selected populations or internal validation, and lack prospective assessment of clinical utility. High discrimination performance should not be interpreted as established clinical utility, because calibration, decision-curve analysis, clinically relevant thresholds, and prospective clinical impact remain insufficiently evaluated. In addition, interpretability and clinical generalizability remain important challenges for translation into routine practice. This review summarizes the current role of CMR in HCM and discusses emerging applications, methodological challenges, and future directions of AI-enhanced CMR for diagnosis, phenotypic characterization, and risk stratification.

## 1. Introduction

Hypertrophic cardiomyopathy (HCM) is one of the most common inherited myocardial diseases and is characterized by otherwise unexplained left ventricular hypertrophy, marked phenotypic heterogeneity, and diverse clinical trajectories [1,2,3]. Although many patients remain clinically stable for years, others may develop atrial fibrillation, progressive heart failure, malignant ventricular arrhythmias, or sudden cardiac death [1,2,3,4]. This wide clinical spectrum, together with overlap between HCM and other causes of left ventricular hypertrophy, continues to challenge accurate diagnosis, phenotypic characterization, individualized risk stratification, and long-term disease management [2,3,4,5].

Cardiac magnetic resonance (CMR) has become a key noninvasive imaging modality for the comprehensive evaluation of HCM [6,7,8]. Owing to its high spatial resolution, unrestricted tomographic coverage, and multiparametric imaging capability, CMR can assess myocardial morphology, ventricular and atrial function, tissue abnormalities, myocardial mechanics, perfusion abnormalities, and disease progression within a single examination [6,7,8,9,10,11,12]. These strengths are particularly important for identifying complex hypertrophy patterns, apical or anterolateral involvement, small ventricular cavities, papillary muscle abnormalities, myocardial crypts, trabecular complexity, apical aneurysm, myocardial fibrosis, diffuse interstitial remodeling, microvascular dysfunction, and functional impairment [9,10,11,12,13,14,15,16,17,18]. Therefore, CMR contributes not only to diagnostic confirmation but also to disease phenotyping, differential diagnosis, treatment planning, follow-up strategy, and prognostic assessment [6,7,8,18,19,20,21,22,23].

Despite these advantages, conventional CMR analysis remains limited by manual or semi-automated workflows, operator dependence, protocol variability, and simplified parameter-based interpretation [24,25,26]. In routine practice, HCM is often summarized using predefined measurements such as maximal wall thickness, ventricular volumes, ejection fraction, left atrial size, late gadolinium enhancement extent, mapping values, perfusion parameters, and strain indices [6,7,8,18,19,20,21,22,23]. These markers are clinically important and remain central to CMR interpretation. However, they may not fully capture the spatial complexity, tissue heterogeneity, scar architecture, dynamic myocardial motion, atrial remodeling, and temporal evolution of HCM. Moreover, multisequence, multiphase, and multiparametric CMR data contain high-dimensional information that is difficult to fully exploit using conventional visual assessment or standard quantitative reporting alone [24,25,26,27].

Artificial intelligence (AI) approaches, particularly machine learning and deep learning, together with radiomics-based quantitative image analysis, provide new opportunities to extend the diagnostic and prognostic value of CMR [28,29,30,31,32]. In cardiovascular imaging, AI has been applied to automated segmentation, functional quantification, tissue characterization, disease identification, risk prediction, dynamic spatiotemporal modeling, image reconstruction, image enhancement, and data augmentation [28,29,32,33,34,35,36,37,38,39,40]. In HCM, these approaches may help extract and integrate quantitative information related to hypertrophy distribution, ventricular geometry, myocardial texture, scar pattern, diffuse tissue abnormalities, myocardial deformation, atrial remodeling, perfusion impairment, and longitudinal disease progression [30,31,33,34,35,40,41,42,43,44,45,46]. Therefore, AI-enhanced CMR may provide a more comprehensive representation of HCM than isolated conventional measurements alone.

Nevertheless, current evidence for AI-enhanced CMR in HCM remains heterogeneous. Although several studies have included independent or external validation, many remain retrospective, single-center, based on selected populations, or limited to internal validation [30,31,33,34,35,40,41,42,43,44,45,46]. Imaging protocols, segmentation strategies, feature extraction methods, model development processes, outcome definitions, and reporting standards vary substantially across studies [24,25,26,27,28,29,32,47,48,49]. In addition, the incremental value of AI-derived CMR signatures over expert interpretation, established diagnostic pathways, and guideline-based risk models has not been consistently demonstrated. Advanced approaches such as dynamic spatiotemporal modeling, generative image synthesis, data augmentation, and foundation models also require further validation regarding biological plausibility, quantitative reliability, interpretability, and clinical safety [28,29,30,31,32].

The added value of the present review is therefore not another general description of the capabilities of AI or CMR. Instead, we use HCM-specific CMR clinical questions as the framework for evaluating the available primary studies. We examine whether computational CMR methods can assist in the diagnosis and differential diagnosis of HCM among patients with otherwise unexplained left ventricular hypertrophy; whether they provide phenotypic information beyond conventional CMR markers; and whether they improve prediction of clinically important outcomes beyond established HCM risk-stratification frameworks. This approach allows the review to address the clinical relevance of each application rather than focusing only on reported model performance.

## 2. Literature Search Strategy

This article was designed as a structured narrative review of artificial intelligence-enhanced cardiac magnetic resonance (CMR) in hypertrophic cardiomyopathy (HCM). The literature search covered publications from January 2005 through to May 2026. PubMed and Scopus were searched using combinations of terms related to hypertrophic cardiomyopathy, cardiac magnetic resonance, artificial intelligence, machine learning, deep learning, radiomics, texture analysis, automated segmentation, automated image analysis, computer-aided methods, and neural networks. The database search was supplemented by manual screening of the reference lists of key primary studies, reviews, clinical guidelines, and consensus statements.

The searches identified 113 records in PubMed and 327 records in Scopus. After removal of 105 duplicate records, 335 unique records remained for title and abstract screening.

Studies were considered relevant if they included patients with HCM or an identifiable HCM subgroup, used CMR or CMR-derived imaging features, and applied artificial intelligence, machine learning, deep learning, radiomics, automated image analysis, automated segmentation, or related computational approaches. Studies involving mixed cardiovascular populations were also considered when their methods or findings were directly relevant to automated, quantitative, or AI-assisted CMR assessment in HCM. Clinical guidelines, consensus statements, methodological studies, and selected reviews were included when necessary to provide clinical or technical context.

Titles and abstracts were screened independently by two reviewers. Full-text assessment and data extraction were also performed independently by two reviewers, with disagreements resolved through discussion and, when necessary, adjudication by a third reviewer.

Table 1 and Table 2 summarize representative HCM-related studies rather than providing an exhaustive systematic-review dataset. Studies were prioritized when they directly addressed the predefined HCM-specific clinical questions, included an identifiable HCM cohort, reported quantitative model performance, or provided independent or external validation. Because this review was intended as a clinically oriented narrative synthesis rather than a systematic review or meta-analysis, no formal quantitative evidence synthesis, PRISMA-based study-selection process, or overall risk-of-bias score was performed.

## 3. CMR Assessment of Hypertrophic Cardiomyopathy: From Imaging Findings to Quantitative Phenotypes

CMR provides a comprehensive framework for evaluating the morphological, functional, hemodynamic, and tissue manifestations of HCM. Although echocardiography remains the first-line imaging modality in most patients, CMR offers high spatial resolution, unrestricted tomographic coverage, and reproducible quantitative assessment, making it particularly valuable when the distribution of hypertrophy is complex, image windows are limited, or additional tissue and functional information is required [6,7,8]. In the context of AI-enhanced imaging, conventional CMR assessment also provides the clinical and quantitative foundation on which automated segmentation, radiomics, machine learning, and deep learning models are developed.

### 3.1. Morphological Assessment and Hypertrophy Patterns

Morphological assessment remains the cornerstone of CMR evaluation in HCM. CMR enables accurate measurement of maximal left ventricular wall thickness, left ventricular mass, chamber size, and the spatial distribution of hypertrophy [6,7,8,9,10]. This is clinically important because HCM may present with asymmetric septal, apical, concentric, mid-ventricular, focal segmental, or mixed hypertrophy patterns. Compared with echocardiography, CMR is particularly useful for detecting apical and anterolateral hypertrophy, where limited acoustic windows or foreshortening may lead to underestimating disease extent [9,10,11,12].

CMR also allows detailed evaluation of structural abnormalities that may influence diagnosis, symptoms, treatment planning, and risk assessment. These include small ventricular cavities, papillary muscle hypertrophy or abnormal insertion, elongated mitral valve leaflets, myocardial crypts, trabecular complexity, mid-ventricular obstruction, and apical aneurysm [10,11,12,13,14,15]. Apical aneurysm is especially important because it may be associated with adverse remodeling, ventricular arrhythmias, thromboembolic events, and increased risk of sudden cardiac death. Therefore, CMR-based morphological assessment should not be limited to confirming wall-thickness criteria but should also define disease distribution and HCM-related structural phenotypes.

### 3.2. Ventricular Function, Atrial Remodeling, and Myocardial Mechanics

Functional assessment in HCM extends beyond left ventricular ejection fraction. Many patients have preserved or supranormal ejection fraction despite substantial myocardial disease, whereas others may develop systolic dysfunction, progressive ventricular dilatation, or end-stage HCM [3,6,7,8]. CMR provides reproducible assessment of ventricular volumes, ejection fraction, left ventricular mass, stroke volume, and remodeling patterns, and is useful for identifying advanced phenotypes such as apical aneurysm, mid-ventricular obstruction, small ventricular cavities, and dilated-phase disease [11,12,13,14,15,16].

Atrial remodeling and myocardial mechanics provide additional information beyond conventional volumetric indices. Left atrial enlargement may reflect chronic diastolic burden, elevated filling pressure, mitral regurgitation, and vulnerability to atrial fibrillation. CMR-derived strain analysis can identify ventricular deformation abnormalities even when ejection fraction is preserved, while left atrial strain may provide insight into atrial myopathy and impaired atrioventricular coupling [17,18,19,20,21,22]. These functional and mechanical abnormalities are clinically relevant because they may be associated with symptoms, exercise intolerance, atrial fibrillation, heart failure progression, and adverse outcomes.

From a quantitative imaging perspective, cine CMR contains dynamic information across the cardiac cycle, but conventional analysis often focuses mainly on end-diastolic and end-systolic frames. Regional motion abnormalities, impaired deformation, dyssynchrony, and atrioventricular interaction may therefore be under-represented in standard reporting. This provides an important rationale for AI-assisted motion analysis and dynamic spatiotemporal modeling in subsequent sections.

### 3.3. Tissue Characterization, Fibrosis, and Microvascular Dysfunction

Tissue characterization is central to CMR assessment of HCM. Late gadolinium enhancement (LGE) can identify replacement fibrosis, which is commonly observed in hypertrophied segments and insertion points and has been associated with adverse clinical outcomes [18,19,20,21,22,23]. However, LGE assessment should not be limited to presence or total extent alone. Scar location, regional distribution, transmural pattern, signal heterogeneity, and progression over time may provide additional information related to disease severity and risk.

Parametric mapping further expands tissue assessment by detecting diffuse interstitial remodeling that may not be visible on LGE images. Native T1 mapping and extracellular volume fraction can reflect diffuse myocardial involvement, extracellular matrix expansion, and early tissue abnormalities, whereas T2 mapping may provide information related to myocardial edema or inflammation in selected settings [23,24,25,26,27]. These parameters may complement LGE by capturing diffuse disease processes beyond focal replacement fibrosis.

Microvascular dysfunction is another clinically relevant feature of HCM and may contribute to ischemia, fibrosis development, symptoms, and disease progression. Stress perfusion CMR and quantitative perfusion approaches can provide information on myocardial blood flow impairment, particularly in patients with severe hypertrophy or microvascular ischemia [50,51,52]. Together, fibrosis, diffuse interstitial remodeling, scar heterogeneity, and perfusion impairment reflect the complex tissue-level phenotype of HCM.

### 3.4. Limitations of Conventional CMR Assessment and Rationale for AI-Based Quantification

Although conventional CMR provides rich diagnostic and prognostic information, its clinical use remains limited by manual or semi-automated post-processing, observer dependence, differences in acquisition protocols, and variability in segmentation and thresholding methods [24,25,26,27]. Manual contouring may be challenging in patients with asymmetric hypertrophy, apical involvement, small ventricular cavities, trabecular complexity, papillary muscle abnormalities, or apical aneurysm. Quantification of LGE extent, mapping values, strain parameters, and perfusion indices may also vary according to software platform, image quality, post-processing workflow, and analytic assumptions.

More importantly, routine CMR interpretation often summarizes HCM using isolated measurements such as maximal wall thickness, ventricular volumes, ejection fraction, left atrial size, LGE extent, mapping values, or strain indices. These markers remain essential, but they may not fully represent the multidimensional nature of HCM. Patients with a similar wall thickness or fibrosis burden may differ substantially in hypertrophy distribution, ventricular geometry, papillary muscle abnormalities, scar architecture, diffuse tissue remodeling, myocardial mechanics, atrial remodeling, perfusion impairment, and longitudinal disease progression.

This gap between the richness of CMR data and the simplicity of conventional reporting provides the rationale for AI-enhanced CMR. Automated segmentation may improve efficiency and reproducibility; radiomics may quantify texture and spatial heterogeneity; machine learning may integrate clinical and imaging variables; deep learning may extract latent imaging patterns from cine, LGE, mapping, or perfusion data; and dynamic spatiotemporal models may exploit motion information across the cardiac cycle. Thus, AI-enhanced CMR should be viewed as an extension of conventional CMR assessment, aiming to transform established imaging markers and high-dimensional image information into reproducible, interpretable, and clinically meaningful HCM phenotypes.

## 4. AI-Enhanced CMR for Diagnosis and Phenotypic Characterization

AI-enhanced CMR has been increasingly investigated as a tool to improve automated image analysis, diagnostic classification, and quantitative phenotypic characterization in HCM. Its value is not limited to replacing manual measurements or classifying images into predefined categories. More importantly, AI may help transform complex CMR information into reproducible imaging signatures that reflect myocardial morphology, hypertrophy distribution, tissue heterogeneity, scar pattern, ventricular mechanics, atrial remodeling, and disease burden. These capabilities are particularly relevant in HCM, where disease expression is spatially heterogeneous and may overlap with other causes of left ventricular hypertrophy. Representative studies applying AI to CMR-based diagnosis and phenotypic characterization in HCM are summarized in Table 1.

**Table 1:** Summary of key studies on AI for CMR-based diagnosis and phenotypic characterization of HCM: methodologies and performance metrics.

Study (1st Author, Year)	Population & Clinical task	CMR Input/Model	Reported Performance	Patient Selection/Reference Standard	Missing Data	Feature Selection/Model Tuning	Validation Independence/Statistical Uncertainty
Neisius U et al. (2019) [34]	HCM 108, HHD 53; HCM vs. HHD	Native T1 radiomics/SVM	Accuracy 86.2% in the feature-selection set and 80.0% in the held-out test set; global T1 accuracy 64.0%.	PS: selected binary comparison; RS: reported	NR	FS: reported; MT: unclear	VI: internal only; SU: partly reported
Zhang X et al. (2023) [40]	HCM 48, DCM 52, controls 123; multiclass classification	CMR radiomics/ML	Overall accuracy up to 91.2%; HCM AUC 0.936.	PS: selected public datasets; RS: unclear	NR	FS: unclear; MT: unclear	VI: no external validation; SU: limited
Pu C et al. (2023) [35]	HCM 273; prediction of LGE positivity	Cine radiomics/XGBoost	Integrated model test AUC 0.898; accuracy 89.02%; sensitivity 92.54%; F1 score 93.23%.	PS: retrospective HCM cohort; RS: expert LGE assessment	NR	FS: unclear; MT: unclear	VI: internal test only; SU: calibration/DCA reported
Zhao K et al. (2024) [41]	HCM *n* = 758; development/internal discovery *n* = 533; external test *n* = 225; MACE events *n* = 191 overall; MACE prediction	Clinical + CMR/ML	Internal AUC 0.830; external AUC 0.812; NRI versus HCM Risk-SCD 0.265.	PS: multicenter clinical cohort; RS/OD: prespecified MACE	NR	FS: yes; MT: yes	VI: external validation; SU: partly reported
Wang YR et al. (2024) [33]	Total *n* = 9719; HCM subgroup *n* = 2715; other CVD and control groups included; multiclass CVD diagnosis including HCM	Cine CMR/Video Swin Transformer	Overall screening AUC 0.988; diagnostic AUC 0.991; HCM AUC 0.998.	PS: large multicenter cohort; RS: expert clinical diagnosis	Excluded incomplete data	FS: NA; MT: reported	VI: external/continuous testing; SU: partly reported
Zhang H et al. (2024) [42]	Total *n* = 621: HCM 421, HHD 200. Random training: validation ratio 6:4; HHD vs. HCM	Cine + LGE radiomics	Combined-model AUC 0.979 in training and 0.981 in validation.	PS: selected binary cohort; RS: explicit criteria	NR	FS: reported; MT: limited	VI: internal only; SU: CIs/calibration reported
Chen WW et al. (2024) [43]	Total *n* = 215: Fabry 156, HCM 59. TCVGH external cohort *n* = 31 (Fabry 20, HCM 11); Fabry vs. HCM	Cine CMR/DL	TVGH AUC 0.914, accuracy 0.909; TCVGH AUC 0.918, accuracy 0.806.	PS: selected phenocopy cohort; RS: reported	NR	FS: NA; MT: CV-based	VI: external validation; SU: limited external precision
Antonopoulos AS et al. (2021) [44]	Analyzed *n* = 149: HCM 61, cardiac amyloidosis 28, other LVH 30, and no heart disease 30; an additional *n* = 20 stability-assessment arm was separate; multiclass classification	Native T1 radiomics/ML	Multinomial C-index 0.753 versus 0.528 for native T1 alone.	PS: small selected phenotype groups; RS: partly reported	NR	FS: whole cohort used before split; MT: CV	VI: internal only; SU: limited
Zhang S et al. (2025) [45]	Total *n* = 253: AL-CA 121, HCM 84, controls 48. Development *n* = 181 (84, 63, 34); hold-out test *n* = 72 (37, 21, 14)	Mapping radiomics/ensemble ML	Test AUC 0.98.	PS: selected multiclass cohort; RS: well-defined	NR	FS: unclear; MT: unclear	VI: same-center hold-out; SU: limited
Fahmy AS et al. (2022) [46]	Final analytic internal cohort *n* = 759: development *n* = 600 and held-out internal test *n* = 159; independent external HCM test *n* = 100. All participants had HCM; LGE positivity prediction	Cine radiomics + DL	Combined-model AUC 0.83 in internal testing and 0.74 in external testing.	PS: multicenter HCM cohort; RS: expert LGE assessment	NR	FS: yes; MT: yes	VI: external validation; SU: external decline reported

**Note:** AL-CA, light-chain cardiac amyloidosis; AUC, area under the receiver operating characteristic curve; C-index, concordance index; CMR, cardiac magnetic resonance; CV, cross-validation; CVD, cardiovascular disease; DCA, decision-curve analysis; DCM, dilated cardiomyopathy; DL, deep learning; HCM, hypertrophic cardiomyopathy; HHD, hypertensive heart disease; LGE, late gadolinium enhancement; MACE, major adverse cardiovascular events; ML, machine learning; SVM, support vector machine; XGBoost, extreme gradient boosting; PS, patient selection; RS, reference standard; OD, outcome definition; FS, feature selection; MT, model tuning; VI, validation independence; SU, statistical uncertainty; NR, not reported; NA, not applicable. Methodological appraisals were based solely on information explicitly reported in the original publications. “Unclear” indicates that the available report did not provide sufficient information to determine the corresponding appraisal item. “Partly” indicates that the criterion was addressed but with identifiable limitations or incomplete reporting. Lack of reporting was not interpreted as evidence of a methodological flaw. A limitation was stated as present only when directly supported by the reported study design or explicitly documented in the original publication. No overall methodological quality or risk-of-bias score was assigned.

### 4.1. Automated Segmentation and Functional Quantification

Automated segmentation is one of the earliest and most established applications of AI in CMR. Accurate delineation of the left ventricular cavity, right ventricular cavity, myocardium, and atrial chambers is essential for measuring ventricular volumes, ejection fraction, myocardial mass, wall thickness, and atrial size. Traditional manual contouring is widely used but is time-consuming and may be affected by observer experience, image quality, and software platform. These limitations are particularly relevant in HCM because asymmetric hypertrophy, apical involvement, small ventricular cavities, trabecular complexity, papillary muscle abnormalities, and apical aneurysm may complicate contour definition.

Deep learning methods, especially convolutional neural network-based models and their variants, have shown strong performance for automated CMR segmentation in large datasets and public benchmark challenges [36,37,38,39,53,54]. Bai et al. demonstrated that a fully convolutional network could automatically segment cardiac chambers and generate clinically relevant functional parameters with high agreement with manual analysis [36]. The ACDC and M&Ms challenges further supported the feasibility of standardized evaluation for automated CMR segmentation and highlighted the importance of multicenter, multi-vendor, and multi-disease validation [53,54]. Although these studies were not restricted to HCM, they provide an important technical foundation for automated CMR workflows.

In HCM-specific or HCM-relevant settings, automated segmentation may improve the efficiency and reproducibility of quantitative assessment. It may facilitate standardized measurement of ventricular volumes, myocardial mass, maximal wall thickness, regional wall-thickness distribution, atrial size, and remodeling patterns. Automated segmentation also provides the basis for downstream radiomics, deformation analysis, motion tracking, and integrated AI-based phenotyping. However, general segmentation models cannot be assumed to perform equally well in HCM patients with severe asymmetric hypertrophy, distorted ventricular geometry, apical aneurysm, or marked trabecular complexity. Visual quality control, uncertainty estimation, and manual correction remain necessary before automated results can be used for clinical decision-making.

AI-assisted functional quantification has also extended from conventional volumetric analysis to myocardial deformation assessment. Deep learning-based segmentation combined with feature-tracking approaches may support automated strain analysis of the left ventricle and left atrium [28,29,37,38]. This is clinically relevant because ventricular and atrial mechanical impairment may be present even when ejection fraction is preserved. In addition, cine CMR contains dynamic information throughout the cardiac cycle, whereas conventional analysis often focuses on end-diastolic and end-systolic frames. Therefore, automated motion analysis and dynamic spatiotemporal modeling may provide additional information on regional motion abnormalities, impaired deformation, dyssynchrony, and atrioventricular interaction.

### 4.2. AI-Assisted Phenotypic Characterization

AI-assisted phenotypic characterization represents an important step beyond automated measurement. Conventional CMR interpretation commonly describes HCM using predefined morphological categories, such as asymmetric septal, apical, concentric, mid-ventricular, focal segmental, or mixed hypertrophy. These categories are clinically useful but may oversimplify the continuous and multidimensional nature of HCM. Patients within the same morphological category may differ substantially in ventricular geometry, wall-thickness distribution, papillary muscle abnormalities, scar pattern, diffuse tissue abnormalities, mechanical impairment, atrial remodeling, and clinical trajectory.

Radiomics and machine learning may help capture these differences by extracting quantitative features related to shape, intensity distribution, texture, and spatial heterogeneity. In HCM, cine-derived radiomic features have been used to characterize myocardial morphology and identify imaging signatures associated with fibrosis, suggesting that non-contrast cine images may contain latent information related to tissue abnormality and disease burden [35]. Tissue-based radiomic approaches using LGE or mapping images may further quantify myocardial heterogeneity, scar architecture, and diffuse remodeling beyond visual assessment or global quantitative markers [30,34,35,44,45].

Deep learning approaches may also contribute to HCM phenotyping by learning imaging representations directly from CMR images. Compared with handcrafted radiomic features, deep learning may capture more complex spatial and temporal patterns, including ventricular shape, hypertrophy distribution, myocardial motion, and tissue-related signal variation. In particular, spatiotemporal models based on cine CMR may provide a framework for evaluating dynamic myocardial motion and deformation patterns across the cardiac cycle. These approaches may be relevant for identifying subtle functional phenotypes, early mechanical impairment, or disease patterns not fully represented by static measurements [28,29,38,43].

The potential clinical value of AI-assisted phenotyping may be greatest in patients with borderline, atypical, or overlapping disease expression. Subtle apical involvement, focal hypertrophy, papillary muscle-related remodeling, regional scar distribution, discordance between preserved ejection fraction and impaired mechanics, or mixed hypertensive and HCM-related features may be difficult to summarize using conventional categories alone. AI-derived imaging phenotypes may therefore complement expert CMR interpretation by providing a more quantitative and reproducible representation of disease patterns. However, no validated AI-based phenotypic classification system has yet been established for routine HCM management, and current studies remain limited by heterogeneity in imaging protocols, feature extraction, model construction, and validation strategies [30,31].

### 4.3. AI-Based Diagnostic Classification and Differential Diagnosis

AI-based diagnostic classification is a clinically relevant application of CMR-derived quantitative imaging features in HCM. Rather than relying on a single measurement such as maximal wall thickness, fibrosis burden, or mapping values, AI models can integrate information related to ventricular morphology, hypertrophy distribution, myocardial texture, scar pattern, diffuse tissue abnormalities, perfusion impairment, and functional remodeling. This approach is important because increased left ventricular wall thickness is not specific to HCM and may also be observed in hypertensive heart disease, athlete’s heart, Fabry cardiomyopathy, cardiac amyloidosis, aortic stenosis, and other infiltrative or metabolic cardiomyopathies.

Compared with broader reviews of artificial intelligence in cardiovascular imaging or HCM, the value of this section lies in its focus on the clinical diagnostic tasks addressed by HCM-specific CMR studies. Rather than summarizing the general ability of AI to classify cardiovascular diseases, we examine whether the reported models address the clinically relevant problem of distinguishing HCM from other causes of otherwise unexplained LVH. We also consider whether the studies used representative clinical populations, clinically appropriate reference standards, and independent validation, and whether their results extend beyond selected binary comparisons.

The distinction between HCM and hypertensive heart disease remains one of the most common diagnostic challenges in patients with left ventricular hypertrophy. Both conditions may show increased wall thickness and myocardial remodeling, and overlap is especially difficult when hypertension coexists with genetically mediated HCM. Neisius et al. demonstrated that radiomic analysis of native T1 mapping could discriminate HCM from hypertensive heart disease, and provided incremental information beyond global native T1 values [34]. More recently, CMR radiomics-based discrimination models using cine and LGE-derived features have shown promising performance for distinguishing HCM from hypertensive heart disease [42]. These findings suggest that AI-enhanced CMR may capture disease-specific myocardial texture, hypertrophy patterns, and fibrosis-related features that are not fully represented by conventional global measurements.

AI-enhanced CMR has also been explored for differentiating HCM from other important phenocopies. Fabry cardiomyopathy may mimic HCM because of left ventricular hypertrophy, but its treatment, genetic counseling, and family screening implications are distinct. Deep learning models using cine CMR have been developed to distinguish Fabry cardiomyopathy from HCM, suggesting that non-contrast images may contain latent morphological and motion-related information useful for phenocopy differentiation [43]. Cardiac amyloidosis is another important mimic of HCM, particularly when increased wall thickness is accompanied by heart failure symptoms or preserved ejection fraction. Machine learning and radiomics approaches based on CMR-derived tissue features have shown potential for distinguishing cardiac amyloidosis from HCM [44,45].

Beyond binary disease comparisons, AI-enhanced CMR may contribute to broader diagnostic workflows for left ventricular hypertrophy and cardiomyopathy classification. Radiomics and deep learning studies have reported the feasibility of classifying HCM, dilated cardiomyopathy, and normal myocardium using CMR-derived features [40]. Multicenter AI-enabled CMR systems have also been developed for automated screening and diagnosis of multiple cardiovascular diseases, with HCM included as one of the major diagnostic categories [33]. These studies provide proof-of-concept evidence that AI-CMR may support scalable disease recognition across heterogeneous cardiovascular populations.

The clinical relevance of these findings depends strongly on the design of the diagnostic comparison. Most available studies compare patients with established HCM with a selected alternative diagnosis or healthy controls. Such studies provide important proof-of-concept evidence that CMR-derived features can distinguish predefined groups, but they do not fully reproduce the clinical evaluation of patients with otherwise unexplained LVH. In routine practice, diagnostic uncertainty may arise from overlapping phenotypes, coexisting hypertension, incomplete disease expression, infiltrative or metabolic disorders, and incomplete genetic or laboratory information. Therefore, the reported performance of these models should not be interpreted as evidence that they can replace comprehensive clinical assessment or resolve the full differential diagnosis of LVH. Further evaluation in representative LVH cohorts, with a prespecified reference standard and independent validation, is required.

## 5. AI-Enhanced CMR for Risk Stratification and Prognostic Assessment

Risk stratification is a central component of HCM management because clinical outcomes vary widely, ranging from long-term stability to atrial fibrillation, progressive heart failure, malignant ventricular arrhythmias, and sudden cardiac death [3,11,55]. Conventional risk assessment incorporates clinical history, family history of sudden cardiac death, unexplained syncope, ventricular arrhythmias, maximal wall thickness, left atrial size, left ventricular outflow tract obstruction, apical aneurysm, systolic dysfunction, and LGE extent [3,12,18,19,55]. Although these markers remain essential, they may not fully capture the complex interactions among hypertrophy distribution, fibrosis burden, scar heterogeneity, diffuse interstitial remodeling, ventricular mechanics, atrial function, and longitudinal disease progression [13,14,15,22,23,56]. AI-enhanced CMR may therefore refine risk prediction by integrating multiparametric imaging information that is often interpreted separately in routine practice. Its value is not simply to generate another risk score but to identify individualized risk-related imaging patterns that better reflect the biological and prognostic complexity of HCM [3,57,58,59]. Representative studies applying AI to CMR-based risk stratification and prognostic assessment in HCM are summarized in Table 2.

**Table 2:** Summary of key studies on artificial intelligence in CMR-based risk stratification and prognostic assessment of HCM.

Study (1st Author, Year)	Population & Clinical Task	CMR Input/Model	Main Performance	Patient Selection/Outcome Definition	Missing Data	Feature Selection/Model Tuning	Validation Independence/Statistical Uncertainty
Zhao K et al. (2024) [41]	HCM *n* = 758; development/internal discovery *n* = 533; external test *n* = 225; MACE *n* = 191 overall; MACE prediction	Clinical + CMR/ML	Internal AUC 0.830; external AUC 0.812; NRI 0.265 versus HCM Risk-SCD.	PS: multicenter cohort; OD: prespecified composite MACE	NR	FS: yes; MT: yes	VI: external; SU: partly reported
Lai C et al. (2025) [60]	Total *n* = 839: internal JHH-HCM *n* = 553 with 19 SCDA events; external SHVI-HCM *n* = 286 with 25 SCDA events. Arrhythmic-event prediction	LGE + multimodal	Internal AUROC 0.89 (95% CI 0.79–0.94); external AUROC 0.81 (95% CI 0.69–0.93); improvement over guideline tools reported.	PS: two independent HCM cohorts; OD: explicitly defined	Imputation reported	FS: NA/prespecified inputs; MT: internal CV	VI: external; SU: bootstrap CIs/Brier reported
Wang J et al. (2021) [61]	HCM *n* = 379; SCD endpoint *n* = 27; no separate development, internal-validation, or external-test cohort reported. SCD prediction	LGE radiomics	C-index 0.834 for the clinical model and 0.852 after adding selected radiomic features.	PS: single-center sequential cohort; OD: defined	NR	FS: internal resampling; MT: internal CV	VI: no external validation; SU: only 27 events
Fahmy AS et al. (2024) [62]	HCM *n* = 1229 from three centers; SCD events *n* = 30; development/internal-validation/external-test allocation not reported. SCD prediction	LGE scar radiomics	C-statistic 0.69 versus 0.57 for ESC and 0.69 versus 0.67 for ACC/AHA; logistic AUC improvements to 0.73 and 0.76 were also reported.	PS: multicenter HCM cohort; OD: defined/adjudicated	Imputation reported	FS: reported; MT: conventional modeling	VI: no external test; SU: only 30 events
Vigneault DM et al. (2017) [38]	Total *n* = 63: HCM 42 and controls 21; no clinical outcome events; no separate development/validation cohort reported	Cine CMR/DL	Pixel accuracy approximately 97%; IoU approximately 87%; circumferential strain differed between HCM and controls.	S: small technical cohort; OD: NA	NR	FS: NA; MT: unclear	VI: internal CV only; SU: prognostic assessment NA

**Note:** AUC, area under the receiver operating characteristic curve; AUROC, area under the receiver operating characteristic curve; C-index, concordance index; CMR, cardiac magnetic resonance; CV, cross-validation; DL, deep learning; HCM, hypertrophic cardiomyopathy; LGE, late gadolinium enhancement; MACE, major adverse cardiovascular events; ML, machine learning; SCD, sudden cardiac death; PS, patient selection; OD, outcome definition; FS, feature selection; MT, model tuning; VI, validation independence; SU, statistical uncertainty; NR, not reported; NA, not applicable. Methodological appraisals were based solely on information explicitly reported in the original publications. “Unclear” indicates that the available report did not provide sufficient information to determine the corresponding appraisal item. “Partly” indicates that the criterion was addressed but with identifiable limitations or incomplete reporting. Lack of reporting was not interpreted as evidence of a methodological flaw. A limitation was stated as present only when directly supported by the reported study design or explicitly documented in the original publication. No overall methodological quality or risk-of-bias score was assigned.

### 5.1. From Isolated CMR Markers to AI-Integrated Risk Phenotypes

CMR-derived prognostic markers in HCM have traditionally been evaluated as individual variables, including maximal wall thickness, apical aneurysm, LGE presence or extent, left ventricular ejection fraction, native T1, extracellular volume, global longitudinal strain, and left atrial strain [16,17,20,21,56]. For example, quantitative LGE has been shown to provide prognostic information for sudden cardiac death risk assessment, while subsequent studies further highlighted the importance of LGE extent, LGE subtype, and serial fibrosis progression [18,19,20,21]. CMR-derived strain parameters, including global longitudinal strain and left atrial strain, have also been associated with adverse outcomes, suggesting that mechanical dysfunction provides complementary prognostic information beyond conventional volumetric indices [13,14,15,16,17,56].

However, HCM prognosis is unlikely to be determined by a single imaging feature. Patients with similar wall thickness may differ in hypertrophy distribution, ventricular geometry, obstruction-related burden, scar distribution, diffuse interstitial remodeling, myocardial deformation, atrial remodeling, and clinical trajectory. Similarly, patients with comparable LGE extent may differ in scar location, signal heterogeneity, tissue composition, and fibrosis progression. These discordant patterns support a shift from isolated imaging markers toward integrated risk profiles that combine morphology, tissue characterization, function, and clinical context.

AI-based approaches may help achieve this integration. Zhao et al. developed an integrative LightGBM model combining clinical and CMR features to predict adverse cardiovascular events in HCM, demonstrating the feasibility of nonlinear risk modeling based on multiparametric information [41]. Lai et al. further developed the MAARS model, which integrated electronic health records, echocardiography and radiology reports, and contrast-enhanced CMR images to forecast lethal arrhythmic events [60]. Radiomics-based studies also support this integrative direction. Wang et al. showed that LGE-derived radiomic features were associated with sudden cardiac death risk in HCM, suggesting that scar texture and spatial organization may contain prognostic information beyond simple LGE quantification [61]. Fahmy et al. subsequently demonstrated in a large multicenter cohort that LGE radiomics, particularly features reflecting scar heterogeneity, provided incremental prognostic value beyond established ESC and AHA/ACC risk models [62]. These studies suggest that AI-integrated risk assessment should be viewed as a strategy for refining, rather than replacing, established clinical frameworks.

### 5.2. AI-Based Tissue Characterization for Risk Prediction

Tissue characterization is a key CMR domain for risk prediction in HCM. LGE reflects replacement fibrosis and has been consistently associated with adverse outcomes, including sudden cardiac death and heart failure-related events [17,19]. Native T1 mapping and extracellular volume quantification further provide markers of diffuse interstitial remodeling, which may be present even in myocardium without visible LGE [22,23,24,25]. However, conventional tissue assessment often summarizes fibrosis using global LGE extent or average mapping values, whereas scar location, spatial distribution, signal heterogeneity, and longitudinal progression may also carry important prognostic information.

LGE radiomics is currently the most developed AI-based approach for tissue characterization in HCM. By extracting features related to scar intensity, texture, shape, and spatial organization, LGE radiomics may provide information beyond conventional fibrosis burden. In a study of 379 patients with HCM and 27 sudden cardiac death events, Wang et al. showed that LGE-derived radiomic features were associated with SCD risk and improved prediction beyond conventional clinical and imaging variables [61]. Fahmy et al. further demonstrated in a multicenter cohort of 1229 patients with HCM that LGE radiomics, particularly features reflecting scar heterogeneity, provided incremental risk stratification beyond established ESC and AHA/ACC risk models [62]. These findings support the concept that the internal architecture of replacement fibrosis, and not merely its total burden, may be clinically relevant.

Mapping-derived markers may also contribute to AI-based tissue risk profiling, although direct prognostic evidence remains less mature than for LGE radiomics. Native T1 and ECV reflect diffuse tissue remodeling, and recent studies have shown their prognostic value in HCM [22,23]. Radiomic analysis of native T1 mapping has also demonstrated the ability to capture myocardial texture information beyond global T1 values in left ventricular hypertrophy phenotypes, suggesting its potential relevance for more detailed tissue characterization [34]. Future AI-CMR models may therefore benefit from integrating LGE-derived scar heterogeneity with mapping-derived diffuse remodeling features, rather than treating replacement fibrosis and interstitial remodeling as separate domains.

Longitudinal tissue characterization is another important direction. Serial myocardial fibrosis assessment has been shown to predict outcomes in HCM, highlighting the prognostic relevance of fibrosis progression rather than baseline fibrosis burden alone [21]. AI-based methods may help quantify subtle interval changes in LGE extent, scar texture, regional fibrosis distribution, native T1, and ECV, thereby supporting dynamic risk prediction. However, tissue-based AI models remain sensitive to acquisition protocols, segmentation strategies, intensity normalization, and scanner-related variability. Future studies should standardize pre-processing pipelines, validate models across centers, and compare AI-derived tissue signatures directly with established markers such as LGE extent, mapping values, apical aneurysm, maximal wall thickness, and guideline-based risk scores.

### 5.3. AI-Based Functional and Deformation Phenotyping

Functional and deformation assessment provides prognostic information that is complementary to morphological and tissue characterization in HCM. Although LVEF is often preserved or supranormal, subtle myocardial dysfunction may already be present and can be detected by CMR feature-tracking-derived strain analysis. Global longitudinal strain has been associated with adverse outcomes in patients with HCM, while regional strain parameters may provide additional prognostic information [16,17]. More recently, feature-tracking-derived global longitudinal strain has been reported to improve sudden cardiac death risk stratification, supporting the incremental value of deformation assessment beyond conventional CMR markers [56].

Left atrial deformation is another important functional marker because atrial dysfunction may reflect chronic diastolic burden, elevated filling pressure, mitral regurgitation, and atrial myopathy. Fast semi-automated left atrial long-axis strain analysis has shown prognostic value in HCM [13]. Subsequent studies further suggested that left atrial strain could predict outcomes after myectomy in obstructive HCM and provide incremental prognostic value in apical HCM [14,15]. These findings indicate that ventricular and atrial mechanics may capture the functional consequences of hypertrophy and fibrosis that are not fully represented by LVEF or chamber size alone.

The role of AI in this area is not merely to calculate strain automatically but to integrate deformation patterns with morphology and tissue characterization. Automated segmentation and motion-tracking algorithms may reduce observer dependence and improve the scalability of deformation analysis. For example, Vigneault et al. combined deep learning-based segmentation with motion tracking to derive circumferential strain automatically in patients with HCM and healthy controls, providing early technical evidence that AI-based workflows can support deformation quantification [38]. In future models, global and regional strain, temporal deformation curves, atrioventricular coupling, LGE characteristics, mapping values, and clinical variables could be jointly analyzed to identify high-risk functional phenotypes.

However, AI-based deformation phenotyping in HCM remains less established than conventional feature-tracking analysis. Strain values vary according to software platform, contouring strategy, temporal resolution, image quality, and vendor-specific algorithms. Moreover, many studies demonstrating the prognostic value of GLS, regional strain, or left atrial strain were not originally designed as AI studies. Future AI-CMR research should therefore validate automated strain extraction across platforms and determine whether AI-derived regional or temporal deformation features provide incremental prognostic value beyond LGE extent, mapping parameters, global strain, left atrial strain, and established clinical risk markers [16,17,28,29,32].

### 5.4. AI-Enabled Longitudinal Assessment and Disease Progression

HCM is a dynamic cardiomyopathy with heterogeneous temporal trajectories, ranging from long-term stability to progressive hypertrophy, fibrosis accumulation, atrial remodeling, and functional deterioration. Conventional CMR is primarily used for cross-sectional evaluation, whereas longitudinal assessment may be limited by inter-study variability in image acquisition, segmentation, and quantitative measurements. In this setting, AI-based approaches may improve the reproducibility and consistency of serial CMR analysis.

Automated segmentation and standardized quantification may reduce variability in the serial assessment of ventricular volumes, wall thickness, LGE extent, mapping values, and strain parameters, thereby facilitating more reliable comparison across follow-up examinations [28,29,32]. Emerging evidence also suggests that serial changes in myocardial fibrosis may carry incremental prognostic information. Tang et al. demonstrated that progression of myocardial fibrosis on serial CMR was associated with adverse outcomes in HCM, highlighting the importance of temporal disease evolution rather than baseline imaging alone [21].

Beyond conventional longitudinal measurements, AI may further enable integrated modeling of disease progression by combining changes in morphology, tissue characteristics, and deformation patterns within unified frameworks. For example, temporal trajectories of wall thickness, LGE heterogeneity, native T1 variation, ECV change, atrial remodeling, and strain deterioration may jointly reflect ongoing myocardial remodeling processes that are not fully captured by single time-point assessments. Such approaches may support earlier identification of high-risk progression patterns and improve the timing of intensified surveillance or therapeutic intervention [63,64,65].

However, AI-based longitudinal modeling in HCM remains at an early stage. Most existing studies are cross-sectional, and only limited evidence is available for fully automated temporal tracking of CMR-derived phenotypes. Key challenges include inter-scan variability, differences in imaging protocols, segmentation consistency over time, and the lack of large longitudinal HCM datasets with repeated CMR examinations and adjudicated outcomes. Future studies should focus on standardized longitudinal pipelines, external validation across centers, and integration with clinical outcomes to determine whether AI-derived temporal features provide incremental value beyond established static risk markers.

## 6. Current Challenges and Limitations

Despite rapid progress, AI-enhanced CMR in HCM remains at an early stage of clinical translation. A major limitation is data availability. HCM is relatively uncommon, and clinically important endpoints such as sudden cardiac death, malignant ventricular arrhythmias, heart failure hospitalization, and thromboembolic events occur infrequently. A substantial proportion of the published literature remains retrospective and single-center, although several studies have incorporated multicenter or external testing [30,31,32,33,34,35,36,37,38,39,40,41,42,43,44,45,46,60,61,62]. This is particularly problematic for radiomics and deep learning models, which involve high-dimensional feature spaces and may be prone to overfitting without robust external validation [28,29,30,31,32].

Imaging heterogeneity further limits reproducibility and generalizability. CMR-derived features may be influenced by scanner vendor, field strength, pulse sequence, contrast administration protocol, spatial resolution, image quality, and post-processing pipeline [24,25,26,27,32,47,48,49]. Radiomic features are sensitive to segmentation strategy, intensity normalization, image pre-processing, and LGE acquisition parameters, whereas mapping values, strain indices, and perfusion parameters may vary according to acquisition settings and software-specific algorithms [13,14,15,16,17,19,20,21,22,23,24,25,26,27,47,48,49,56,57,58]. These sources of variability remain major obstacles to cross-center application.

Segmentation and annotation variability also affect downstream AI outputs. Differences in contouring ventricular borders, trabeculations, papillary muscles, scar regions, or atrial borders may influence radiomic features, deformation indices, and predictive models [24,25,26,27,32,36,37,38,39,47,48,49]. Although fully automated segmentation can improve efficiency, errors may be amplified in HCM because of asymmetric hypertrophy, small ventricular cavities, apical involvement, apical aneurysm, and trabecular complexity. Robust quality control, uncertainty estimation, and transparent reporting of segmentation performance are therefore essential.

Interpretability is another key challenge. Although AI models may achieve favorable diagnostic or prognostic performance, clinicians need to understand which imaging regions, features, or disease patterns contribute to model outputs. This is especially important in HCM, where AI-derived predictions may influence ICD consideration, rhythm monitoring, anticoagulation, follow-up intensity, genetic counseling, and referral for septal reduction or advanced therapies [1,3,55]. Explainable AI approaches, including feature attribution, regional risk mapping, attention visualization, and phenotype-based interpretation, may help improve clinical trust and biological plausibility [28,29,43,60].

Moreover, many studies emphasize discrimination metrics without demonstrating clinical utility. High AUC or C-index values do not necessarily translate into improved decision-making or better patient outcomes. Future validation should include calibration, decision-curve analysis, reclassification metrics, incremental value over guideline-based models, and real-world clinical impact [28,29,30,31,32]. Models should also be evaluated according to specific clinical tasks, because diagnosis, phenocopy differentiation, sudden cardiac death prediction, heart failure risk assessment, and longitudinal disease monitoring may require different features, endpoints, and validation strategies.

The methodological reliability of the available studies is heterogeneous and should be considered at the level of individual studies rather than as a uniform limitation of the entire field. The principal appraisal domains include patient selection, the clinical relevance and quality of the reference standard, handling of missing data, feature selection, model tuning, independence of validation, and statistical uncertainty.

Taken together, the principal studies do not share the same methodological profile. Some provide direct HCM evidence with independent testing, whereas others are based on internal validation, selected case–control comparisons, or limited numbers of clinical events. Several reports do not provide sufficient information to determine how missing data, feature selection, or model tuning were handled. These uncertainties should not be interpreted as confirmed flaws, but they limit the strength of the conclusions that can be drawn from the available evidence. The current literature therefore supports a graded interpretation: some applications have more credible validation, whereas others remain exploratory or provide mainly technical support for future HCM-specific studies.

## 7. Future Perspectives

Future development of AI-enhanced CMR in HCM will depend on larger datasets, methodological innovation, and clinical integration. A key priority is the establishment of large-scale, multicenter, and well-phenotyped HCM cohorts with standardized imaging protocols and harmonized outcome definitions. Such datasets are needed to capture diverse HCM phenotypes, including apical HCM, obstructive and non-obstructive disease, apical aneurysm, dilated-phase HCM, early genotype-positive disease, and phenocopy-like presentations. They are also essential for modeling clinically important but infrequent outcomes, such as sudden cardiac death, malignant ventricular arrhythmias, heart failure hospitalization, and thromboembolic events.

Emerging learning strategies may help overcome current limitations in labeled data and model generalizability. Self-supervised learning, weakly supervised learning, transfer learning, and federated learning may reduce dependence on large manually annotated datasets and support multicenter model development while preserving data privacy. Foundation models trained on large-scale CMR datasets may provide adaptable representations for segmentation, diagnosis, phenotyping, and risk prediction. However, these approaches require HCM-specific validation because complex hypertrophy, small ventricular cavities, papillary muscle abnormalities, apical aneurysm, scar heterogeneity, and protocol variability may challenge general-purpose models [28,29,30,31,32].

Future AI-CMR research should also make better use of dynamic and multimodal information. Dynamic spatiotemporal modeling of cine CMR may capture regional motion abnormalities, mechanical dyssynchrony, impaired relaxation, atrioventricular coupling, and subtle functional deterioration that are not fully represented by static measurements. Multimodal AI frameworks integrating cine imaging, LGE, mapping, perfusion, strain, clinical variables, electrocardiography, echocardiography, genetic information, biomarkers, and longitudinal follow-up data may provide more comprehensive representations of HCM biology and risk [28,29,43,60].

AI-assisted reconstruction, image enhancement, and data augmentation may further improve CMR efficiency and model development. Deep learning reconstruction may shorten acquisition time, while generative models and synthetic data may help augment imaging datasets for rare HCM phenotypes. Importantly, synthetic images may augment model development but cannot substitute for independent cohorts of real patients with observed clinical outcomes when evaluating prognostic generalizability. Nevertheless, these approaches must be carefully validated because enhanced or synthetic images may alter myocardial borders, scar texture, signal intensity distribution, mapping values, or radiomic features. For HCM, where diagnosis and risk assessment often depend on subtle differences in wall thickness, fibrosis extent, and tissue heterogeneity, technical improvements should be evaluated according to quantitative reliability and clinical interpretability [28,29,30,31,32]. Synthetic images may support model development and technical robustness assessment, but they cannot substitute for an independent cohort of real patients with observed clinical outcomes when evaluating prognostic generalizability.

Ultimately, AI-enhanced CMR should aim not only to improve diagnostic or predictive accuracy but also to support clinically meaningful decision-making. Explainable AI methods, uncertainty estimation, and clinically interpretable outputs will be essential for integration into routine reporting, multidisciplinary decision-making, and longitudinal follow-up. Prospective multicenter studies are needed to determine whether AI-CMR can improve diagnostic confidence, risk reclassification, ICD decision-making, rhythm monitoring, follow-up intensity, therapeutic planning, and patient outcomes. With continued validation and workflow integration, AI-enhanced CMR may evolve from a research tool into a practical decision-support system for individualized HCM management [28,29,30,31,32,60].

## 8. Conclusions

CMR plays a central role in the comprehensive evaluation of HCM by enabling detailed assessment of myocardial morphology, ventricular and atrial function, tissue characterization, myocardial deformation, perfusion abnormalities, and disease progression. AI-enhanced CMR has the potential to extend this value by improving automated image analysis, quantitative phenotypic characterization, diagnostic classification, differentiation from HCM phenocopies, and individualized risk prediction.

Current evidence suggests that radiomics, machine learning, deep learning, and emerging dynamic and multimodal approaches may help capture complex imaging patterns related to hypertrophy distribution, scar heterogeneity, diffuse tissue remodeling, mechanical impairment, atrial remodeling, and longitudinal disease evolution. These approaches may provide complementary information beyond conventional CMR measurements and support more individualized disease assessment. However, the strength of the available evidence is heterogeneous. Some studies provide direct HCM-specific evidence with independent or external validation, whereas others remain exploratory because they rely on selected populations, internal validation, limited outcome events, or predefined binary comparisons. Studies that primarily evaluate automated image processing or derive findings from broader cardiovascular cohorts provide methodological or technical support but should not be interpreted as direct evidence of clinical benefit in HCM.

The clinical translation of AI-enhanced CMR therefore remains at an early stage. High AUC or C-index values demonstrate statistical discrimination but do not by themselves establish incremental clinical utility beyond expert CMR interpretation or established HCM risk-stratification frameworks. Calibration, decision-curve analysis, reclassification, clinically relevant thresholds, independent external validation, and prospective effects on clinical management and patient outcomes remain insufficiently evaluated. These limitations are particularly important when considering the potential use of AI-derived CMR information for individualized risk assessment or other management decisions.

Future progress will require large and clinically representative multicenter cohorts, standardized imaging and analytical pipelines, transparent reporting of model development and validation, reproducible and interpretable outputs, and direct comparison with established CMR markers and clinical risk models. With such evidence, AI-enhanced CMR may become a useful adjunct for precision imaging and clinical decision support in HCM; however, current findings support further validation rather than routine clinical implementation.

## Data Availability

No new data were created or analyzed in this study. Data sharing is not applicable to this article.
